# 1,10-Bis(4-nitro­phen­oxy)deca­ne

**DOI:** 10.1107/S1600536810003855

**Published:** 2010-02-06

**Authors:** Toheed Akhter, Humaira M. Siddiqi, Zareen Akhter, Michael Bolte

**Affiliations:** a Department of Chemistry, Quaid-I-Azam University, Islamabad 45320, Pakistan; b Institut für Anorganische Chemie, J. W. Goethe-Universität Frankfurt, Max-von-Laue-Strasse 7, 60438 Frankfurt/Main, Germany

## Abstract

The title compound, C_22_H_28_N_2_O_6_, crystallizes with four half-mol­ecules in the asymmetric unit: each mol­ecule is located about a crystallographic inversion centre. The central methyl­ene groups of two mol­ecules are disordered over two sets of equally occupied sites. The crystal packing is characterized by sheets of mol­ecules parallel to (1
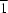
4).

## Related literature

For background to thermally-stable polyimides, see: Hsiao & Leu (2004 ▶). Hsiao *et al.* (2004 ▶); Mehdipour-Ataei (2005 ▶); Mehdipour-Ataei *et al.* (2006 ▶); Schab-Balcerzak *et al.* (2002 ▶).
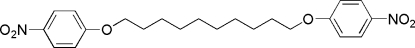


## Experimental

### 

#### Crystal data

C_22_H_28_N_2_O_6_
*M* *_r_* = 416.47
Triclinic, 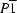

*a* = 10.1806 (10) Å
*b* = 14.9460 (13) Å
*c* = 16.1127 (15) Å
α = 114.370 (7)°
β = 94.322 (8)°
γ = 103.861 (8)°
*V* = 2125.7 (3) Å^3^
*Z* = 4
Mo *K*α radiation
μ = 0.10 mm^−1^
*T* = 173 K
0.27 × 0.14 × 0.14 mm

#### Data collection

Stoe IPDS II two-circle diffractometer
24686 measured reflections
7975 independent reflections
3974 reflections with *I* > 2σ(*I*)
*R*_int_ = 0.097

#### Refinement

*R*[*F* ^2^ > 2σ(*F* ^2^)] = 0.054
*wR*(*F* ^2^) = 0.132
*S* = 0.83
7975 reflections
559 parameters
8 restraints
H-atom parameters constrained
Δρ_max_ = 0.33 e Å^−3^
Δρ_min_ = −0.26 e Å^−3^

### 

Data collection: *X-AREA* (Stoe & Cie, 2001 ▶); cell refinement: *X-AREA*; data reduction: *X-AREA*; program(s) used to solve structure: *SHELXS97* (Sheldrick, 2008 ▶); program(s) used to refine structure: *SHELXL97* (Sheldrick, 2008 ▶); molecular graphics: *XP* in *SHELXTL* (Sheldrick, 2008 ▶); software used to prepare material for publication: *SHELXL97*.

## Supplementary Material

Crystal structure: contains datablocks I, global. DOI: 10.1107/S1600536810003855/tk2623sup1.cif

Structure factors: contains datablocks I. DOI: 10.1107/S1600536810003855/tk2623Isup2.hkl

Additional supplementary materials:  crystallographic information; 3D view; checkCIF report

## Figures and Tables

**Fig. 1.**
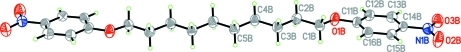
A view of one of the four molecules in the unit cell of the title compound, with the atom-numbering scheme. Displacement ellipsoids are drawn at the 50% probability level and H atoms are shown as small spheres of arbitrary radii. Only the symmetry independent atoms are labelled. Symmetry operator for the generated atoms -x+2, -y+1, -z.

## supplementary crystallographic information

### Comment

The title dinitro compound is a precursor for aromatic diamines which are important compounds for the synthesis of thermally-stable polyimides (Mehdipour-Ataei, 2005). Polyimides are an important class of high-performance polymers having useful properties such as excellent thermal and mechanical strength, good solvent resistance and adhesion to glass and metals (Mehdipour-Ataei *et al.*, 2006). However, applications of polyimides are limited because they are difficult to process due to their reduced solubility in organic solvents. Solubility problems arise due to chain stiffness and intermolecular hydrogen bonding (Hsiao and Leu, 2004). The title compound was synthesized with the aim to design new monomers containing an aryl-ether linkage for processable high-performance polyimides which contain methylene spacers between the aromatic rings connected by an ether moiety. The aryl-ether linkage is well known for imparting properties such as improved solubility and melt processing characteristics (Hsiao *et al.*, 2004). Moreover, the introduction of long flexible aliphatic chains into the aromatic backbone will effectively disrupt the intermolecular interactions between the aromatic moieties responsible for their high glass transition (Tg) temperatures (Schab-Balcerzak *et al.*, 2002).

The title compound crystallizes with four half molecules in the asymmetric unit, see Fig. 1 for a representative molecule. Each molecule is located about a crystallographic inversion centre. The central methylene groups of two molecules are disordered over two equally occupied sites. The crystal packing is characterized by sheets of molecules parallel to the (1 1 4) plane.

### Experimental

A three-necked round bottom flask equipped with thermometer, magnetic stirrer and nitrogen inlet was charged with a suspension of *p*-nitrophenol (5 g, 35 mol) and anhydrous potassium carbonate (5 g, 0.035 mol) in N,N'-dimethyl formamide (DMF; 60 ml) and stirred (at room temperature) for 1 h. After that solution of 1, 10-dibromodecane (5.2 g, 70 mol) in DMF (10 ml) was added drop wise and the mixture was refluxed for 16 h at 120°C. The progress of reaction was monitored by TLC [ethyl acetate: n-hexane, 1:4]. After the reaction completed, reaction mixture was poured into distilled water (500 ml) which led to precipitation of the product as a solid yellow mass. The crude product was filtered, washed thoroughly with water, dissolved in ethanol, and set aside for crystallization. Yield 86%, m.p. 351-352 K.

### Refinement

Hydrogen atoms were included in calculated positions [C—H = 0.95Å or 0.99 Å] and refined as riding [U_iso_(H) = 1.2U_eq_(C)]. The central methylene groups of two molecules are disordered over two equally occupied sites. Bond distances involving the disordered C atoms were restrained to 1.50 (1) Å.

### Crystal data

**Table:**

C_22_H_28_N_2_O_6_	*Z* = 4
*M**_r_* = 416.47	*F*(000) = 888
Triclinic, *P*1	*D*_x_ = 1.301 Mg m^−^^3^
Hall symbol: -P 1	Mo *K*α radiation, λ = 0.71073 Å
*a* = 10.1806 (10) Å	Cell parameters from 9860 reflections
*b* = 14.9460 (13) Å	θ = 3.3–25.8°
*c* = 16.1127 (15) Å	µ = 0.10 mm^−^^1^
α = 114.370 (7)°	*T* = 173 K
β = 94.322 (8)°	Block, colourless
γ = 103.861 (8)°	0.27 × 0.14 × 0.14 mm
*V* = 2125.7 (3) Å^3^	

### Data collection

**Table:**

Stoe IPDS II two-circle diffractometer	3974 reflections with *I* > 2σ(*I*)
Radiation source: fine-focus sealed tube	*R*_int_ = 0.097
graphite	θ_max_ = 25.8°, θ_min_ = 3.2°
ω scans	*h* = −12→12
24686 measured reflections	*k* = −18→18
7975 independent reflections	*l* = −19→19

### Refinement

**Table:**

Refinement on *F*^2^	Primary atom site location: structure-invariant direct methods
Least-squares matrix: full	Secondary atom site location: difference Fourier map
*R*[*F*^2^ > 2σ(*F*^2^)] = 0.054	Hydrogen site location: inferred from neighbouring sites
*wR*(*F*^2^) = 0.132	H-atom parameters constrained
*S* = 0.83	*w* = 1/[σ^2^(*F*_o_^2^) + (0.056*P*)^2^] where *P* = (*F*_o_^2^ + 2*F*_c_^2^)/3
7975 reflections	(Δ/σ)_max_ < 0.001
559 parameters	Δρ_max_ = 0.33 e Å^−^^3^
8 restraints	Δρ_min_ = −0.26 e Å^−^^3^

### Special details

**Table:**

Geometry. All esds (except the esd in the dihedral angle between two l.s. planes) are estimated using the full covariance matrix. The cell esds are taken into account individually in the estimation of esds in distances, angles and torsion angles; correlations between esds in cell parameters are only used when they are defined by crystal symmetry. An approximate (isotropic) treatment of cell esds is used for estimating esds involving l.s. planes.
Refinement. Refinement of *F*^2^ against ALL reflections. The weighted *R*-factor wR and goodness of fit *S* are based on *F*^2^, conventional *R*-factors *R* are based on *F*, with *F* set to zero for negative *F*^2^. The threshold expression of *F*^2^ > σ(*F*^2^) is used only for calculating *R*-factors(gt) etc. and is not relevant to the choice of reflections for refinement. *R*-factors based on *F*^2^ are statistically about twice as large as those based on *F*, and *R*- factors based on ALL data will be even larger.

### Fractional atomic coordinates and isotropic or equivalent isotropic displacement parameters (Å^2^)

**Table:**

	*x*	*y*	*z*	*U*_iso_*/*U*_eq_	Occ. (<1)
N1A	−0.577 (11)	−0.282 (8)	0.186 (7)	0.06 (3)	
O1A	−0.076 (8)	−0.096 (6)	0.125 (6)	0.05 (2)	
O2A	−0.604 (10)	−0.371 (7)	0.175 (7)	0.07 (3)	
O3A	−0.658 (10)	−0.231 (7)	0.209 (7)	0.08 (3)	
C1A	0.019 (12)	−0.153 (8)	0.086 (9)	0.05 (3)	
H1A1	−0.0270	−0.2118	0.0250	0.058*	
H1A2	0.0526	−0.1791	0.1279	0.058*	
C2A	0.137 (12)	−0.078 (8)	0.075 (9)	0.05 (3)	
H2A1	0.1805	−0.0193	0.1361	0.060*	
H2A2	0.1012	−0.0518	0.0337	0.060*	
C3A	0.247 (12)	−0.127 (9)	0.034 (9)	0.05 (3)	
H3A1	0.2022	−0.1925	−0.0228	0.063*	
H3A2	0.2942	−0.1436	0.0792	0.063*	
C4A	0.354 (15)	−0.056 (11)	0.009 (12)	0.08 (5)	
H4A1	0.3992	−0.1004	−0.0364	0.096*	0.50
H4A2	0.3017	−0.0294	−0.0251	0.096*	0.50
H4A3	0.3038	−0.0339	−0.0305	0.096*	0.50
H4A4	0.4027	0.0061	0.0670	0.096*	0.50
C5A'	0.46 (3)	0.029 (17)	0.075 (18)	0.07 (7)	0.50
H5AA	0.5185	0.0030	0.1063	0.081*	0.50
H5AB	0.4158	0.0718	0.1223	0.081*	0.50
C5A"	0.45 (2)	−0.095 (18)	−0.04 (2)	0.06 (8)	0.50
H5AC	0.4046	−0.1624	−0.0906	0.076*	0.50
H5AD	0.5133	−0.1080	0.0054	0.076*	0.50
C11A	−0.196 (12)	−0.147 (8)	0.138 (8)	0.04 (3)	
C12A	−0.284 (12)	−0.088 (8)	0.175 (8)	0.05 (3)	
H12A	−0.2573	−0.0168	0.1883	0.058*	
C13A	−0.407 (12)	−0.131 (8)	0.191 (8)	0.05 (3)	
H13A	−0.4666	−0.0901	0.2163	0.056*	
C14A	−0.445 (12)	−0.234 (8)	0.171 (8)	0.04 (3)	
C15A	−0.359 (12)	−0.294 (8)	0.135 (8)	0.05 (3)	
H15A	−0.3858	−0.3642	0.1219	0.055*	
C16A	−0.235 (12)	−0.250 (8)	0.118 (8)	0.05 (3)	
H16A	−0.1766	−0.2911	0.0931	0.055*	
N1B	−0.103 (9)	0.250 (7)	0.215 (7)	0.04 (2)	
O1B	0.402 (7)	0.295 (5)	0.106 (6)	0.05 (2)	
O2B	−0.155 (9)	0.321 (6)	0.237 (7)	0.07 (3)	
O3B	−0.160 (9)	0.169 (6)	0.216 (6)	0.06 (2)	
C1B	0.487 (11)	0.390 (8)	0.111 (8)	0.04 (3)	
H1B1	0.5087	0.4450	0.1751	0.051*	
H1B2	0.4384	0.4112	0.0697	0.051*	
C2B	0.617 (11)	0.369 (8)	0.079 (8)	0.04 (3)	
H2B1	0.6684	0.3551	0.1248	0.053*	
H2B2	0.5912	0.3058	0.0189	0.053*	
C3B	0.711 (11)	0.455 (8)	0.067 (8)	0.04 (3)	
H3B1	0.7392	0.5181	0.1268	0.053*	
H3B2	0.6605	0.4696	0.0212	0.053*	
C4B	0.841 (11)	0.430 (8)	0.033 (8)	0.04 (3)	
H4B1	0.8930	0.4180	0.0800	0.052*	
H4B2	0.8127	0.3658	−0.0254	0.052*	
C5B	0.937 (11)	0.513 (9)	0.017 (9)	0.05 (3)	
H5B1	0.9668	0.5778	0.0760	0.055*	
H5B2	0.8850	0.5265	−0.0291	0.055*	
C11B	0.279 (11)	0.291 (8)	0.132 (8)	0.04 (3)	
C12B	0.208 (11)	0.197 (8)	0.127 (8)	0.04 (3)	
H12B	0.2461	0.1415	0.1042	0.051*	
C13B	0.084 (11)	0.182 (8)	0.154 (8)	0.04 (3)	
H13B	0.0361	0.1175	0.1509	0.052*	
C14B	0.030 (10)	0.265 (8)	0.187 (7)	0.04 (2)	
C15B	0.097 (10)	0.359 (7)	0.192 (7)	0.04 (3)	
H15B	0.0565	0.4137	0.2140	0.044*	
C16B	0.224 (11)	0.374 (8)	0.165 (8)	0.04 (3)	
H16B	0.2716	0.4387	0.1680	0.048*	
N1C	−0.017 (12)	−0.386 (7)	0.286 (7)	0.05 (3)	
O1C	−0.112 (8)	−0.007 (5)	0.396 (6)	0.05 (2)	
O2C	0.091 (10)	−0.397 (6)	0.260 (7)	0.07 (3)	
O3C	−0.106 (10)	−0.454 (6)	0.290 (7)	0.07 (3)	
C1C	−0.019 (11)	0.080 (7)	0.392 (8)	0.04 (3)	
H1C1	−0.0067	0.0639	0.3277	0.049*	
H1C2	0.0725	0.1002	0.4317	0.049*	
C2C	−0.084 (11)	0.167 (7)	0.428 (8)	0.04 (3)	
H2C1	−0.1068	0.1755	0.4894	0.050*	
H2C2	−0.1720	0.1472	0.3849	0.050*	
C3C	0.009 (11)	0.268 (7)	0.438 (8)	0.04 (3)	
H3C1	0.0337	0.2588	0.3778	0.047*	
H3C2	0.0949	0.2889	0.4834	0.047*	
C4C	−0.059 (11)	0.355 (8)	0.472 (8)	0.04 (3)	
H4C1	−0.1454	0.3342	0.4267	0.050*	
H4C2	−0.0844	0.3643	0.5323	0.050*	
C5C	0.033 (11)	0.457 (7)	0.482 (8)	0.04 (3)	
H5C1	0.0557	0.4476	0.4211	0.049*	
H5C2	0.1202	0.4762	0.5257	0.049*	
C11C	−0.081 (11)	−0.097 (7)	0.366 (8)	0.04 (3)	
C12C	−0.181 (12)	−0.176 (8)	0.369 (8)	0.04 (3)	
H12C	−0.2631	−0.1653	0.3890	0.053*	
C13C	−0.161 (12)	−0.272 (8)	0.342 (8)	0.04 (3)	
H13C	−0.2277	−0.3270	0.3444	0.054*	
C14C	−0.041 (12)	−0.285 (8)	0.311 (8)	0.04 (3)	
C15C	0.059 (11)	−0.208 (8)	0.307 (8)	0.04 (3)	
H15C	0.1403	−0.2202	0.2858	0.051*	
C16C	0.039 (11)	−0.112 (8)	0.334 (8)	0.04 (3)	
H16C	0.1055	−0.0573	0.3308	0.050*	
N1D	0.410 (12)	0.130 (7)	0.309 (8)	0.06 (3)	
O1D	0.524 (8)	0.540 (6)	0.377 (6)	0.05 (2)	
O2D	0.298 (10)	0.086 (6)	0.317 (7)	0.07 (3)	
O3D	0.500 (12)	0.088 (7)	0.285 (9)	0.10 (4)	
C1D	0.427 (11)	0.597 (8)	0.409 (8)	0.04 (3)	
H1D1	0.4106	0.6005	0.4703	0.053*	
H1D2	0.3379	0.5627	0.3645	0.053*	
C2D	0.492 (12)	0.703 (8)	0.418 (9)	0.05 (3)	
H2D1	0.5810	0.7357	0.4621	0.061*	
H2D2	0.5101	0.6977	0.3564	0.061*	
C3D	0.398 (14)	0.771 (8)	0.450 (10)	0.06 (4)	
H3D1	0.3826	0.7790	0.5126	0.072*	
H3D2	0.3076	0.7375	0.4071	0.072*	
C4D	0.46 (2)	0.877 (11)	0.455 (13)	0.10 (6)	
H4D1	0.5542	0.9077	0.4948	0.118*	0.50
H4D2	0.4717	0.8684	0.3920	0.118*	0.50
H4D3	0.4967	0.8659	0.3975	0.118*	0.50
H4D4	0.3830	0.9058	0.4519	0.118*	0.50
C5D'	0.39 (3)	0.947 (17)	0.49 (3)	0.08 (10)	0.50
H5DA	0.3902	0.9646	0.5557	0.095*	0.50
H5DB	0.2905	0.9123	0.4560	0.095*	0.50
C5D"	0.56 (3)	0.95 (2)	0.52 (2)	0.08 (9)	0.50
H5DC	0.6381	0.9256	0.5307	0.092*	0.50
H5DD	0.5235	0.9691	0.5829	0.092*	0.50
C11D	0.490 (11)	0.440 (8)	0.364 (8)	0.04 (3)	
C12D	0.586 (11)	0.388 (8)	0.329 (8)	0.04 (3)	
H12D	0.6686	0.4227	0.3167	0.053*	
C13D	0.560 (11)	0.287 (8)	0.311 (8)	0.04 (3)	
H13D	0.6240	0.2507	0.2871	0.052*	
C14D	0.440 (11)	0.238 (8)	0.329 (8)	0.04 (3)	
C15D	0.345 (11)	0.289 (8)	0.365 (8)	0.04 (3)	
H15D	0.2637	0.2547	0.3772	0.051*	
C16D	0.370 (11)	0.391 (8)	0.382 (8)	0.04 (3)	
H16D	0.3062	0.4264	0.4065	0.049*	

### Atomic displacement parameters (Å^2^)

**Table:**

	*U*^11^	*U*^22^	*U*^33^	*U*^12^	*U*^13^	*U*^23^
N1A	0.06 (7)	0.06 (7)	0.05 (7)	0.01 (5)	0.01 (5)	0.02 (5)
O1A	0.05 (5)	0.04 (4)	0.07 (6)	0.02 (4)	0.02 (4)	0.02 (4)
O2A	0.08 (7)	0.06 (6)	0.08 (7)	0.01 (5)	0.02 (5)	0.04 (5)
O3A	0.06 (6)	0.08 (6)	0.09 (8)	0.03 (5)	0.04 (6)	0.04 (6)
C1A	0.05 (7)	0.05 (6)	0.05 (8)	0.02 (6)	0.01 (6)	0.02 (6)
C2A	0.05 (7)	0.04 (6)	0.05 (8)	0.01 (5)	0.01 (6)	0.02 (5)
C3A	0.05 (7)	0.05 (6)	0.06 (8)	0.01 (5)	0.02 (6)	0.02 (6)
C4A	0.06 (9)	0.08 (10)	0.09 (12)	0.02 (8)	0.02 (9)	0.04 (9)
C5A'	0.07 (18)	0.07 (16)	0.06 (19)	0.03 (14)	0.02 (15)	0.02 (14)
C5A"	0.05 (15)	0.05 (14)	0.1 (2)	0.02 (12)	0.03 (15)	0.04 (15)
C11A	0.05 (7)	0.04 (6)	0.04 (7)	0.01 (5)	0.01 (5)	0.02 (5)
C12A	0.05 (7)	0.04 (6)	0.05 (8)	0.02 (5)	0.01 (6)	0.02 (6)
C13A	0.05 (7)	0.04 (6)	0.05 (8)	0.02 (5)	0.01 (6)	0.02 (5)
C14A	0.05 (7)	0.05 (6)	0.04 (7)	0.02 (5)	0.01 (6)	0.02 (5)
C15A	0.06 (8)	0.04 (6)	0.04 (7)	0.02 (5)	0.01 (6)	0.02 (5)
C16A	0.05 (7)	0.04 (6)	0.05 (7)	0.02 (5)	0.01 (6)	0.02 (5)
N1B	0.04 (5)	0.04 (5)	0.04 (6)	0.01 (4)	0.01 (4)	0.02 (4)
O1B	0.04 (4)	0.05 (4)	0.07 (6)	0.02 (4)	0.02 (4)	0.03 (4)
O2B	0.06 (5)	0.06 (5)	0.10 (8)	0.03 (4)	0.04 (5)	0.04 (5)
O3B	0.05 (5)	0.05 (5)	0.07 (6)	0.01 (4)	0.02 (4)	0.03 (4)
C1B	0.04 (6)	0.04 (6)	0.05 (7)	0.01 (5)	0.01 (5)	0.02 (5)
C2B	0.04 (6)	0.05 (6)	0.05 (7)	0.02 (5)	0.01 (5)	0.02 (6)
C3B	0.04 (6)	0.04 (6)	0.05 (7)	0.02 (5)	0.02 (5)	0.02 (5)
C4B	0.04 (6)	0.04 (6)	0.05 (8)	0.02 (5)	0.02 (5)	0.02 (5)
C5B	0.04 (6)	0.05 (6)	0.06 (8)	0.02 (5)	0.02 (6)	0.03 (5)
C11B	0.03 (6)	0.05 (6)	0.04 (7)	0.02 (5)	0.01 (5)	0.02 (5)
C12B	0.04 (6)	0.04 (6)	0.05 (7)	0.02 (5)	0.01 (5)	0.02 (5)
C13B	0.05 (7)	0.03 (5)	0.05 (8)	0.02 (5)	0.01 (6)	0.02 (5)
C14B	0.03 (6)	0.04 (6)	0.04 (6)	0.02 (5)	0.01 (5)	0.02 (5)
C15B	0.04 (6)	0.03 (5)	0.04 (7)	0.02 (5)	0.01 (5)	0.01 (5)
C16B	0.04 (6)	0.04 (5)	0.04 (7)	0.01 (5)	0.01 (5)	0.02 (5)
N1C	0.06 (7)	0.04 (5)	0.06 (7)	0.02 (5)	0.01 (6)	0.02 (5)
O1C	0.05 (5)	0.03 (4)	0.06 (5)	0.01 (3)	0.02 (4)	0.02 (4)
O2C	0.07 (6)	0.05 (5)	0.10 (8)	0.03 (5)	0.02 (6)	0.03 (5)
O3C	0.08 (7)	0.04 (4)	0.09 (7)	0.01 (4)	0.02 (5)	0.03 (4)
C1C	0.04 (6)	0.03 (5)	0.04 (7)	0.01 (5)	0.01 (5)	0.02 (5)
C2C	0.04 (6)	0.04 (5)	0.04 (7)	0.01 (5)	0.01 (5)	0.02 (5)
C3C	0.04 (6)	0.04 (6)	0.04 (7)	0.02 (5)	0.01 (5)	0.02 (5)
C4C	0.04 (6)	0.04 (6)	0.05 (7)	0.01 (5)	0.01 (5)	0.02 (5)
C5C	0.04 (6)	0.04 (5)	0.05 (7)	0.01 (5)	0.01 (5)	0.02 (5)
C11C	0.04 (6)	0.03 (5)	0.04 (7)	0.01 (5)	0.01 (5)	0.02 (5)
C12C	0.04 (7)	0.04 (6)	0.05 (7)	0.01 (5)	0.01 (5)	0.02 (5)
C13C	0.05 (7)	0.04 (6)	0.04 (7)	0.01 (5)	0.01 (6)	0.02 (5)
C14C	0.05 (7)	0.03 (5)	0.04 (7)	0.01 (5)	0.01 (5)	0.02 (5)
C15C	0.04 (6)	0.04 (6)	0.05 (7)	0.02 (5)	0.01 (5)	0.02 (5)
C16C	0.05 (6)	0.03 (5)	0.05 (7)	0.01 (5)	0.01 (5)	0.02 (5)
N1D	0.06 (7)	0.05 (6)	0.06 (8)	0.01 (5)	0.02 (6)	0.02 (5)
O1D	0.05 (5)	0.05 (4)	0.06 (6)	0.02 (4)	0.02 (4)	0.03 (4)
O2D	0.06 (6)	0.05 (5)	0.10 (8)	0.00 (5)	0.02 (5)	0.03 (5)
O3D	0.09 (8)	0.05 (5)	0.14 (11)	0.04 (5)	0.06 (7)	0.03 (6)
C1D	0.04 (6)	0.05 (6)	0.05 (7)	0.02 (5)	0.01 (5)	0.02 (5)
C2D	0.05 (7)	0.05 (6)	0.05 (8)	0.01 (5)	0.01 (6)	0.03 (6)
C3D	0.06 (8)	0.04 (6)	0.08 (10)	0.01 (6)	0.01 (7)	0.03 (6)
C4D	0.13 (15)	0.05 (8)	0.10 (14)	0.01 (9)	0.02 (12)	0.02 (9)
C5D'	0.07 (18)	0.03 (12)	0.1 (3)	0.03 (12)	0.05 (19)	0.04 (16)
C5D"	0.1 (2)	0.1 (2)	0.1 (2)	0.05 (18)	0.02 (17)	0.03 (18)
C11D	0.04 (6)	0.05 (6)	0.04 (6)	0.01 (5)	0.01 (5)	0.02 (5)
C12D	0.04 (6)	0.05 (7)	0.04 (7)	0.01 (5)	0.02 (5)	0.02 (6)
C13D	0.04 (6)	0.05 (6)	0.04 (7)	0.02 (5)	0.01 (5)	0.01 (5)
C14D	0.04 (6)	0.04 (6)	0.04 (7)	0.01 (5)	0.01 (5)	0.01 (5)
C15D	0.03 (6)	0.05 (6)	0.04 (7)	0.01 (5)	0.01 (5)	0.02 (5)
C16D	0.03 (6)	0.05 (6)	0.05 (7)	0.02 (5)	0.01 (5)	0.02 (5)

### Geometric parameters (Å, °)

**Table:**

N1A—O3A	1.23 (13)	N1C—O3C	1.23 (13)
N1A—O2A	1.24 (13)	N1C—O2C	1.22 (13)
N1A—C14A	1.45 (15)	N1C—C14C	1.47 (13)
O1A—C11A	1.35 (13)	O1C—C11C	1.36 (12)
O1A—C1A	1.45 (13)	O1C—C1C	1.44 (12)
C1A—C2A	1.51 (16)	C1C—C2C	1.52 (13)
C1A—H1A1	0.9900	C1C—H1C1	0.9900
C1A—H1A2	0.9900	C1C—H1C2	0.9900
C2A—C3A	1.52 (16)	C2C—C3C	1.52 (14)
C2A—H2A1	0.9900	C2C—H2C1	0.9900
C2A—H2A2	0.9900	C2C—H2C2	0.9900
C3A—C4A	1.53 (18)	C3C—C4C	1.53 (13)
C3A—H3A1	0.9900	C3C—H3C1	0.9900
C3A—H3A2	0.9900	C3C—H3C2	0.9900
C4A—C5A'	1.4 (2)	C4C—C5C	1.52 (14)
C4A—C5A"	1.4 (2)	C4C—H4C1	0.9900
C4A—H4A1	0.9900	C4C—H4C2	0.9900
C4A—H4A2	0.9900	C5C—C5C^ii^	1.52 (19)
C4A—H4A3	0.9900	C5C—H5C1	0.9900
C4A—H4A4	0.9900	C5C—H5C2	0.9900
C5A'—H5AA	0.9900	C11C—C12C	1.39 (15)
C5A'—H5AB	0.9900	C11C—C16C	1.39 (15)
C5A"—H5AC	0.9900	C12C—C13C	1.38 (14)
C5A"—H5AD	0.9900	C12C—H12C	0.9500
C11A—C16A	1.38 (15)	C13C—C14C	1.38 (16)
C11A—C12A	1.41 (15)	C13C—H13C	0.9500
C12A—C13A	1.37 (16)	C14C—C15C	1.37 (15)
C12A—H12A	0.9500	C15C—C16C	1.39 (14)
C13A—C14A	1.39 (15)	C15C—H15C	0.9500
C13A—H13A	0.9500	C16C—H16C	0.9500
C14A—C15A	1.39 (15)	N1D—O2D	1.22 (13)
C15A—C16A	1.37 (16)	N1D—O3D	1.23 (13)
C15A—H15A	0.9500	N1D—C14D	1.46 (14)
C16A—H16A	0.9500	O1D—C11D	1.36 (13)
N1B—O3B	1.23 (11)	O1D—C1D	1.45 (12)
N1B—O2B	1.23 (11)	C1D—C2D	1.52 (15)
N1B—C14B	1.46 (13)	C1D—H1D1	0.9900
O1B—C11B	1.35 (12)	C1D—H1D2	0.9900
O1B—C1B	1.43 (12)	C2D—C3D	1.52 (16)
C1B—C2B	1.52 (14)	C2D—H2D1	0.9900
C1B—H1B1	0.9900	C2D—H2D2	0.9900
C1B—H1B2	0.9900	C3D—C4D	1.53 (18)
C2B—C3B	1.51 (15)	C3D—H3D1	0.9900
C2B—H2B1	0.9900	C3D—H3D2	0.9900
C2B—H2B2	0.9900	C4D—C5D"	1.3 (3)
C3B—C4B	1.53 (14)	C4D—C5D'	1.4 (2)
C3B—H3B1	0.9900	C4D—H4D1	0.9900
C3B—H3B2	0.9900	C4D—H4D2	0.9900
C4B—C5B	1.51 (15)	C4D—H4D3	0.9900
C4B—H4B1	0.9900	C4D—H4D4	0.9900
C4B—H4B2	0.9900	C5D'—H5DA	0.9900
C5B—C5B^i^	1.5 (2)	C5D'—H5DB	0.9900
C5B—H5B1	0.9900	C5D"—H5DC	0.9900
C5B—H5B2	0.9900	C5D"—H5DD	0.9899
C11B—C12B	1.38 (15)	C11D—C16D	1.39 (15)
C11B—C16B	1.41 (14)	C11D—C12D	1.40 (15)
C12B—C13B	1.36 (15)	C12D—C13D	1.37 (15)
C12B—H12B	0.9500	C12D—H12D	0.9500
C13B—C14B	1.39 (13)	C13D—C14D	1.38 (15)
C13B—H13B	0.9500	C13D—H13D	0.9500
C14B—C15B	1.37 (14)	C14D—C15D	1.39 (15)
C15B—C16B	1.39 (15)	C15D—C16D	1.37 (15)
C15B—H15B	0.9500	C15D—H15D	0.9500
C16B—H16B	0.9500	C16D—H16D	0.9500
			
O3A—N1A—O2A	123 (10)	O3C—N1C—C14C	118 (10)
O3A—N1A—C14A	118 (10)	O2C—N1C—C14C	118 (10)
O2A—N1A—C14A	119 (10)	C11C—O1C—C1C	119 (8)
C11A—O1A—C1A	119 (8)	O1C—C1C—C2C	106 (8)
O1A—C1A—C2A	107 (8)	O1C—C1C—H1C1	110.6
O1A—C1A—H1A1	110.4	C2C—C1C—H1C1	110.6
C2A—C1A—H1A1	110.4	O1C—C1C—H1C2	110.6
O1A—C1A—H1A2	110.4	C2C—C1C—H1C2	110.6
C2A—C1A—H1A2	110.4	H1C1—C1C—H1C2	108.7
H1A1—C1A—H1A2	108.6	C1C—C2C—C3C	113 (9)
C1A—C2A—C3A	113 (9)	C1C—C2C—H2C1	109.1
C1A—C2A—H2A1	109.0	C3C—C2C—H2C1	109.1
C3A—C2A—H2A1	109.0	C1C—C2C—H2C2	109.1
C1A—C2A—H2A2	109.0	C3C—C2C—H2C2	109.1
C3A—C2A—H2A2	109.0	H2C1—C2C—H2C2	107.8
H2A1—C2A—H2A2	107.8	C2C—C3C—C4C	113 (9)
C2A—C3A—C4A	112 (10)	C2C—C3C—H3C1	109.0
C2A—C3A—H3A1	109.2	C4C—C3C—H3C1	109.0
C4A—C3A—H3A1	109.2	C2C—C3C—H3C2	109.0
C2A—C3A—H3A2	109.2	C4C—C3C—H3C2	109.0
C4A—C3A—H3A2	109.2	H3C1—C3C—H3C2	107.8
H3A1—C3A—H3A2	107.9	C5C—C4C—C3C	113 (9)
C5A'—C4A—C3A	123 (10)	C5C—C4C—H4C1	108.9
C5A"—C4A—C3A	118 (10)	C3C—C4C—H4C1	108.9
C5A'—C4A—H4A1	106.5	C5C—C4C—H4C2	108.9
C3A—C4A—H4A1	106.5	C3C—C4C—H4C2	108.9
C5A'—C4A—H4A2	106.5	H4C1—C4C—H4C2	107.8
C5A"—C4A—H4A2	112.7	C4C—C5C—C5C^ii^	113 (10)
C3A—C4A—H4A2	106.5	C4C—C5C—H5C1	108.9
H4A1—C4A—H4A2	106.5	C5C^ii^—C5C—H5C1	108.9
C5A'—C4A—H4A3	109.3	C4C—C5C—H5C2	108.9
C5A"—C4A—H4A3	107.9	C5C^ii^—C5C—H5C2	108.9
C3A—C4A—H4A3	107.9	H5C1—C5C—H5C2	107.7
H4A1—C4A—H4A3	101.2	O1C—C11C—C12C	114 (9)
C5A"—C4A—H4A4	107.9	O1C—C11C—C16C	125 (9)
C3A—C4A—H4A4	107.9	C12C—C11C—C16C	121 (9)
H4A1—C4A—H4A4	125.2	C13C—C12C—C11C	120 (10)
H4A2—C4A—H4A4	103.0	C13C—C12C—H12C	119.9
H4A3—C4A—H4A4	107.2	C11C—C12C—H12C	119.9
C4A—C5A'—H5AA	107.9	C14C—C13C—C12C	118 (10)
C4A—C5A'—H5AB	107.0	C14C—C13C—H13C	121.0
H5AA—C5A'—H5AB	107.6	C12C—C13C—H13C	121.0
C4A—C5A"—H5AC	107.3	C15C—C14C—C13C	123 (9)
C4A—C5A"—H5AD	108.3	C15C—C14C—N1C	119 (10)
H5AC—C5A"—H5AD	107.5	C13C—C14C—N1C	118 (10)
O1A—C11A—C16A	125 (10)	C14C—C15C—C16C	119 (10)
O1A—C11A—C12A	115 (9)	C14C—C15C—H15C	120.5
C16A—C11A—C12A	119 (10)	C16C—C15C—H15C	120.5
C13A—C12A—C11A	120 (10)	C11C—C16C—C15C	119 (10)
C13A—C12A—H12A	119.8	C11C—C16C—H16C	120.6
C11A—C12A—H12A	119.8	C15C—C16C—H16C	120.6
C12A—C13A—C14A	119 (10)	O2D—N1D—O3D	124 (10)
C12A—C13A—H13A	120.4	O2D—N1D—C14D	119 (10)
C14A—C13A—H13A	120.4	O3D—N1D—C14D	118 (10)
C13A—C14A—C15A	121 (10)	C11D—O1D—C1D	118 (8)
C13A—C14A—N1A	120 (10)	O1D—C1D—C2D	106 (9)
C15A—C14A—N1A	119 (10)	O1D—C1D—H1D1	110.5
C16A—C15A—C14A	120 (10)	C2D—C1D—H1D1	110.5
C16A—C15A—H15A	120.1	O1D—C1D—H1D2	110.5
C14A—C15A—H15A	120.1	C2D—C1D—H1D2	110.5
C15A—C16A—C11A	120 (10)	H1D1—C1D—H1D2	108.7
C15A—C16A—H16A	119.9	C3D—C2D—C1D	111 (10)
C11A—C16A—H16A	119.9	C3D—C2D—H2D1	109.3
O3B—N1B—O2B	123 (10)	C1D—C2D—H2D1	109.3
O3B—N1B—C14B	119 (9)	C3D—C2D—H2D2	109.3
O2B—N1B—C14B	118 (9)	C1D—C2D—H2D2	109.3
C11B—O1B—C1B	120 (8)	H2D1—C2D—H2D2	108.0
O1B—C1B—C2B	106 (8)	C2D—C3D—C4D	112 (10)
O1B—C1B—H1B1	110.5	C2D—C3D—H3D1	109.3
C2B—C1B—H1B1	110.5	C4D—C3D—H3D1	109.3
O1B—C1B—H1B2	110.5	C2D—C3D—H3D2	109.3
C2B—C1B—H1B2	110.5	C4D—C3D—H3D2	109.3
H1B1—C1B—H1B2	108.7	H3D1—C3D—H3D2	108.0
C3B—C2B—C1B	114 (9)	C5D"—C4D—C5D'	82 (10)
C3B—C2B—H2B1	108.7	C5D"—C4D—C3D	123 (10)
C1B—C2B—H2B1	108.7	C5D'—C4D—C3D	116 (10)
C3B—C2B—H2B2	108.7	C5D'—C4D—H4D1	108.2
C1B—C2B—H2B2	108.7	C3D—C4D—H4D1	108.2
H2B1—C2B—H2B2	107.6	C5D"—C4D—H4D2	115.9
C2B—C3B—C4B	113 (9)	C5D'—C4D—H4D2	108.2
C2B—C3B—H3B1	109.1	C3D—C4D—H4D2	108.2
C4B—C3B—H3B1	109.1	H4D1—C4D—H4D2	107.4
C2B—C3B—H3B2	109.1	C5D"—C4D—H4D3	106.5
C4B—C3B—H3B2	109.1	C5D'—C4D—H4D3	121.5
H3B1—C3B—H3B2	107.8	C3D—C4D—H4D3	106.5
C5B—C4B—C3B	114 (8)	H4D1—C4D—H4D3	93.2
C5B—C4B—H4B1	108.6	C5D"—C4D—H4D4	106.5
C3B—C4B—H4B1	108.6	C3D—C4D—H4D4	106.5
C5B—C4B—H4B2	108.6	H4D1—C4D—H4D4	132.8
C3B—C4B—H4B2	108.6	H4D2—C4D—H4D4	90.7
H4B1—C4B—H4B2	107.6	H4D3—C4D—H4D4	106.5
C4B—C5B—C5B^i^	114 (10)	C4D—C5D'—H5DA	108.4
C4B—C5B—H5B1	108.9	C4D—C5D'—H5DB	108.2
C5B^i^—C5B—H5B1	108.9	H5DA—C5D'—H5DB	107.7
C4B—C5B—H5B2	108.9	C4D—C5D"—H5DC	108.4
C5B^i^—C5B—H5B2	108.9	C4D—C5D"—H5DD	108.2
H5B1—C5B—H5B2	107.7	H5DC—C5D"—H5DD	107.5
O1B—C11B—C12B	116 (9)	O1D—C11D—C16D	125 (9)
O1B—C11B—C16B	124 (10)	O1D—C11D—C12D	115 (9)
C12B—C11B—C16B	120 (10)	C16D—C11D—C12D	121 (10)
C13B—C12B—C11B	122 (10)	C13D—C12D—C11D	119 (10)
C13B—C12B—H12B	119.1	C13D—C12D—H12D	120.4
C11B—C12B—H12B	119.1	C11D—C12D—H12D	120.4
C12B—C13B—C14B	118 (10)	C12D—C13D—C14D	119 (10)
C12B—C13B—H13B	121.1	C12D—C13D—H13D	120.3
C14B—C13B—H13B	121.1	C14D—C13D—H13D	120.3
C15B—C14B—C13B	122 (10)	C13D—C14D—C15D	122 (10)
C15B—C14B—N1B	119 (9)	C13D—C14D—N1D	120 (10)
C13B—C14B—N1B	119 (9)	C15D—C14D—N1D	118 (10)
C14B—C15B—C16B	120 (9)	C16D—C15D—C14D	119 (10)
C14B—C15B—H15B	120.2	C16D—C15D—H15D	120.4
C16B—C15B—H15B	120.2	C14D—C15D—H15D	120.4
C15B—C16B—C11B	118 (9)	C15D—C16D—C11D	120 (10)
C15B—C16B—H16B	120.9	C15D—C16D—H16D	120.1
C11B—C16B—H16B	120.9	C11D—C16D—H16D	120.1
O3C—N1C—O2C	124 (9)		
			
C11A—O1A—C1A—C2A	177 (10)	C11C—O1C—C1C—C2C	179 (10)
O1A—C1A—C2A—C3A	−180 (10)	O1C—C1C—C2C—C3C	174 (9)
C1A—C2A—C3A—C4A	171 (11)	C1C—C2C—C3C—C4C	178 (10)
C2A—C3A—C4A—C5A'	77 (21)	C2C—C3C—C4C—C5C	180 (10)
C2A—C3A—C4A—C5A"	−174 (19)	C3C—C4C—C5C—C5C^ii^	−178 (12)
C1A—O1A—C11A—C16A	1(17)	C1C—O1C—C11C—C12C	−177 (10)
C1A—O1A—C11A—C12A	−179 (11)	C1C—O1C—C11C—C16C	2(17)
O1A—C11A—C12A—C13A	−180 (11)	O1C—C11C—C12C—C13C	−179 (10)
C16A—C11A—C12A—C13A	0(18)	C16C—C11C—C12C—C13C	2(18)
C11A—C12A—C13A—C14A	0(18)	C11C—C12C—C13C—C14C	−1(17)
C12A—C13A—C14A—C15A	0(18)	C12C—C13C—C14C—C15C	1(18)
C12A—C13A—C14A—N1A	−179 (11)	C12C—C13C—C14C—N1C	178 (10)
O3A—N1A—C14A—C13A	6(17)	O3C—N1C—C14C—C15C	180 (11)
O2A—N1A—C14A—C13A	−174 (11)	O2C—N1C—C14C—C15C	−1(17)
O3A—N1A—C14A—C15A	−173 (12)	O3C—N1C—C14C—C13C	3(17)
O2A—N1A—C14A—C15A	7(17)	O2C—N1C—C14C—C13C	−178 (11)
C13A—C14A—C15A—C16A	−1(18)	C13C—C14C—C15C—C16C	−1(18)
N1A—C14A—C15A—C16A	178 (10)	N1C—C14C—C15C—C16C	−178 (11)
C14A—C15A—C16A—C11A	1(18)	O1C—C11C—C16C—C15C	179 (11)
O1A—C11A—C16A—C15A	179 (11)	C12C—C11C—C16C—C15C	−2(17)
C12A—C11A—C16A—C15A	0(18)	C14C—C15C—C16C—C11C	1(17)
C11B—O1B—C1B—C2B	178 (10)	C11D—O1D—C1D—C2D	180 (10)
O1B—C1B—C2B—C3B	172 (10)	O1D—C1D—C2D—C3D	179 (10)
C1B—C2B—C3B—C4B	−179 (10)	C1D—C2D—C3D—C4D	−178 (13)
C2B—C3B—C4B—C5B	178 (10)	C2D—C3D—C4D—C5D"	−79 (25)
C3B—C4B—C5B—C5B^i^	−179 (13)	C2D—C3D—C4D—C5D'	−177 (22)
C1B—O1B—C11B—C12B	−178 (10)	C1D—O1D—C11D—C16D	−2(16)
C1B—O1B—C11B—C16B	1(16)	C1D—O1D—C11D—C12D	177 (10)
O1B—C11B—C12B—C13B	179 (11)	O1D—C11D—C12D—C13D	−179 (10)
C16B—C11B—C12B—C13B	−1(18)	C16D—C11D—C12D—C13D	1(17)
C11B—C12B—C13B—C14B	1(18)	C11D—C12D—C13D—C14D	0(17)
C12B—C13B—C14B—C15B	0(17)	C12D—C13D—C14D—C15D	0(18)
C12B—C13B—C14B—N1B	179 (10)	C12D—C13D—C14D—N1D	179 (11)
O3B—N1B—C14B—C15B	−175 (10)	O2D—N1D—C14D—C13D	−173 (11)
O2B—N1B—C14B—C15B	5(15)	O3D—N1D—C14D—C13D	7(18)
O3B—N1B—C14B—C13B	5(16)	O2D—N1D—C14D—C15D	6(17)
O2B—N1B—C14B—C13B	−175 (11)	O3D—N1D—C14D—C15D	−174 (12)
C13B—C14B—C15B—C16B	0(17)	C13D—C14D—C15D—C16D	1(18)
N1B—C14B—C15B—C16B	−180 (10)	N1D—C14D—C15D—C16D	−179 (11)
C14B—C15B—C16B—C11B	0(16)	C14D—C15D—C16D—C11D	0(17)
O1B—C11B—C16B—C15B	−179 (10)	O1D—C11D—C16D—C15D	179 (10)
C12B—C11B—C16B—C15B	1(16)	C12D—C11D—C16D—C15D	0(17)

Symmetry codes: (i) −*x*+2, −*y*+1, −*z*; (ii) −*x*, −*y*+1, −*z*+1.
